# Supplementary material for: Robust β‐Sheet Peptide Reinforced Polymer Fibers
Source: Small Sci. 2025 May 26;5(8):2500115. doi: 10.1002/smsc.202500115 (PMC12362809; doi:10.1002/smsc.202500115)
Supplement: Supplementary file 1 — Supplementary Material [file SMSC-5-2500115-s001.pdf]

Robust β-sheet peptide reinforced polymer fibers

Nicholas J. Chan, Sarah Lentz, Paul A. Gurr, Shereen Tan, Mona Schultebeyring, Sabine Rosenfeldt, Anna Schenk, Thomas Scheibel, Greg G. Qiao**

N. J. Chan, S. Lentz, P. A. Gurr, S. Tan, G. G. Qiao
Polymer Science Group, Department of Chemical Engineering
University of Melbourne
Parkville, Melbourne, Victoria 3010, Australia
Email: gregghq@unimelb.edu.au

N. J. Chan, S. Lentz, M. Schultebeyring, T. Scheibel
Lehrstuhl Biomaterialien
Universität Bayreuth
Prof.-Rüdiger-Bormann-Str. 1, D-95447 Bayreuth, Germany
Email: thomas.scheibel@uni-bayreuth.de

T. Scheibel
Bayreuther Zentrum für Kolloide und Grenzflächen (BZKG)
University of Bayreuth
Universitätsstraße 30, D-95447 Bayreuth, Germany

T. Scheibel
Bayreuther Zentrum für Molekulare Biowissenschaften (BZMB)
University of Bayreuth
Universitätsstraße 30, D-95447 Bayreuth, Germany

T. Scheibel
Bayreuther Materialzentrum (BayMAT)
University of Bayreuth
Universitätsstraße 30, D-95447 Bayreuth, Germany

S. Rosenfeldt, A. Schenk, T. Scheibel
Bavarian Polymer Institute (BPI)

Universität Bayreuth
D-95447 Bayreuth, Germany

S. Rosenfeldt
Physical Chemistry 1
Universität Bayreuth
D-95447 Bayreuth, Germany

A. Schenk
Physical Chemistry - Colloidal Systems
Universität Bayreuth
D-95447 Bayreuth, Germany

Keywords: NCA ROP, crystallization, spider silk, Nylon, cellulose

In natural silks, β-sheet crystals are embedded within an amorphous matrix resulting in polypeptide-based nanocomposites. These β-sheet crystals contribute to the subsequent high strength and toughness of spider silk. Consequently, imitation and mimicry of such concepts utilizing polypeptides provides a pathway towards putatively achieving similar properties. Herein, we investigate the introduction of poly(L-valine) (PVal) β-sheet nanocrystals into different fibers. Analysis of micro- and nanoscale features showed that polyvaline β-sheets could be implemented into fibers made from different polymer classes, ranging from standard polymers (polycaprolactone (PCL), Nylon 6) to biopolymers like cellulose and recombinant spider silk. The in-situ implementation of PVal during wet-spinning led to a significant change in the resulting mechanical properties, depending on the polymer used.

1. Introduction

Nature provides a near infinite number of blueprints for the bioinspiration of robust synthetic materials.[1, 2] At the forefront of these are materials derived from proteins, which experience various intra- and intermolecular interactions such as hydrogen bonding and van der Waals forces. The foundation of any higher order structure is within the underlying secondary structures – including α-helices and β-sheets.[3, 4] Both secondary structures are rigid and contribute to an increase in material strength due to their lattice packing structures and

subsequent intermolecular interactions between individual proteins. Intramolecular crystal formation results from the coiled-coil and lamellae structures in the case of α-helices and βsheets, respectively.[5] However, intermolecular stacks of these secondary structures are often brittle on their own. Thus, the use of nanocomposite structures provides an avenue to yield tough fibrous materials where such rigid structures exist within a more amorphous continuous phase. One prime example is the dragline spider silk with a high tensile strength comparable to high tensile steel, but with orders of magnitude higher toughness.[6-9] The underlying spider silk proteins consist of two major categories of repetitive motifs; i) short poly(L-alanine) segments (approximately 5-8 residues in length), which form rigid β-sheet nanocrystals and ii) glycinerich segments resulting in less-ordered structures including random coils. Upon tensile load on the silk fibers, energy is dissipated between the β-sheet nanocrystals within the less-ordered semi-amorphous matrix, before aligning and eventually unravelling prior to rupture.[10-12] The net result is a fiber with a tensile strength between 0.88–1.5 GPa and an extension at break of 21–27 % depending on the silk type and spider species.[13] As such, the fabrication of composite fibers with a peptidic component capable of forming rigid structures responsible for superior mechanical properties, such as β-sheets and synthetic polymeric components similar to spidersilk, pose great potential.[14-16]

Multiple strategies have focused on the design and optimization of composites comprising polymers and β-sheet forming peptides.[17] The general design strategy for nanocomposite polymeric materials involves introducing nanoparticles (such as graphene-based crystalline nanoparticles, nanocellulose or clays) into the solution as a dispersion within a polymeric matrix.[18-20] In the case of polypeptide composites, the polypeptide often forms a 3-D matrix instead of individual nanoparticle components.[21] Polypeptide nanoparticle introduction into a polymeric matrix has been well researched, but primarily as a micelle for payload delivery,[22-25] rather than explicitly for mechanical reinforcement. The development of discrete β-sheet nanostructures for reinforcement is largely complicated due to uncontrolled aggregation, although strategies to control this such as templating with other small molecules[26, 27] or unique fabrication techniques are beginning to develop.[28] Polypeptide sequences are attached to a polymer either through coupling or the use of a polymer as a macroinitiator, to influence βsheet formation.[29-31] The resulting microphase separation during material fabrication yields the

desired composite on a mesoscale. ABA triblock polymer conformations are particularly popular for such work with a central soft synthetic polymer flanked by peptidic components.[32, 33]

The interactions between different components of nanocomposite fibers are an important factor for their subsequent mechanical properties, though further complicated by self-assembly systems triggering inherent changes of both the polymer and the peptidic component.[34-37] Interactions between the peptide and the non-peptidic component inherently affects the structure formation of both polymers.[38, 39] The studies of the Korley group on films developed from ABA triblock copolymers exemplify this combined morphological impact.[40-44] This influence extends to the other non-peptidic component, such as affecting crystallinity and spherulite formation specifically in poly(ethylene glycol) (PEG) upon introduction of poly(Cbz-L-lysine).[45] As poly(Cbz-L-lysine) was introduced, hydrogen bonding between PEG and peptide bonds resulted in the disruption of intermolecular bonds of PEG, leading to reduced crystallinity. The secondary structure was found to have some degree of impact as well, as samples containing β-sheets were found to have lower crystallinity than their α-helical counterparts at the same mass concentration. The dehydration of PEG molecules upon heating in PEG-poly(alanine) hydrogels resulted in tighter packing of the conjugates and, thus, induced β-sheet formation.[46, 47] Such design traits, while universally useful, do not perfectly translate between different material morphologies, with microfibers being a particularly interesting morphology.

For industrial applications, such as aircraft or textile technologies, one-step processes to obtain significantly reinforced and sustainable biopolymer-based microfibers without an additional matrix are increasingly demanded. Potential candidates are polypeptide-based fibers which primarily utilize recombinant or purified natural structural proteins as the sole component of the fiber. While keratin, collagen and casein also have been utilized for such artificial materials,[48-51] spider silk forms the basis of many works where either synthetic mimetics or subtle derivatives are investigated. Regenerated silk fibroin derived from silkworms (*i.e. Bombyx mori* spider silk) offers a close natural facsimile, sharing similar polypeptide sequence motifs with spider silk despite not exhibiting the same mechanical superiority.[52-54] Fibers spun

from recombinant spider silk proteins follow a pathway to achieving similar mechanical properties in comparison to natural spider silk.[55, 56]

Our approach includes the reinforcement of well-established microfibers by introducing βsheets using *N*-carboxyanhydride ring opening polymerization (NCA ROP). Although NCA ROP is amenable to high throughput production of polypeptides, its application in polypeptide based microfibers is insufficiently investigated.[57] To date, we have found only two others studies which have explored the use of NCA ROP derived polypeptides within a microfiber.[58, 59] However, both strategies required molecular changes to the polymer yielding multiple step processes to even synthesize and purify the desired polymer. Thus, a more facile approach to polypeptide integration is desirable. Building on our previous published work which utilized βsheet forming polypeptides, derived from NCA ROP poly-L-valine (PVal), with increased strength, we seek to extend this concept in the formation of microfibers.[60]

Herein, we present a novel and broadly applicable technique for introducing β-sheet forming polypeptides into wet spun microfibers from unmodified polymers for physical reinforcement by the introduction of L-valine NCA (Val NCA) monomer into the spinning dope and thus controlling the subsequent β-sheet formation. Through this method, we have presented a novel methodology of inducing polymer crystallization through β-sheet formation. The investigation includes various classes of polymers: Nylon 6 and poly(caprolactone) (PCL) (synthetic), Cellulose and cellulose acetate (polysaccharide) and poly(benzyl-L-glutamate) (PBLG) and eADF4(C16) (polypeptide and protein). Interestingly, the change in the mechanical properties of the reinforced fibers is linked to the change in the underlying morphological changes.

2. Results and Discussion

Wet spinning was utilized to fabricate fibers by dissolving NCA and polymer within the spinning dope and extruding the solution into a coagulation bath designed to initiate NCA ROP and produce a β-sheet forming polypeptide within the fiber (Figure 1a, b). The solvent/nonsolvent system was designed such that polymerization would only occur upon extrusion into the coagulation bath. As such, anhydrous solvents or a mixture of anhydrous and acidic solvents were utilized in the spinning dope, and 2 % v/v triethylamine (TEA) was introduced into the coagulation bath to initiate polymerization. Nonsolvent coagulation baths containing diethyl ether were initially tested but were found to cause the polymers to precipitate too quickly to form continuous fibers. Thus, the nonsolvent coagulation baths of a high fraction

of water (or even 100% water) were utilized, which normally would cause the indiscriminate ring opening of NCA. However, the inclusion of a small amount of TEA, as in our case, was recently shown by Heise *et al.* to successfully initiate NCA ROP within an oil/water emulsion with successful polymerization instead of individual ring-opened monomers.[61] By utilizing TEA as a tertiary amine initiator, NCA ROP occurs *via* the faster, but uncontrolled activated monomer mechanism (AMM) instead of the slower normal amine NCA ROP mechanism usually associated with the use of primary amine initiators.[62, 63] Heise *et al.* hypothesized that the faster polymerization route mitigated the effect of indiscriminate ROP *via* water in their colloidal system and thus was applied to this system such that polymerization would occur as fast as possible once extruded into coagulation baths containing water. Val NCA was utilized as monomer as its homopolypeptide counterpart, PVal, has a high β-sheet forming propensity (especially compared to high molecular weight poly(L-alanine)) in an effort to induce more favorable mechanical properties as was established in our previous work.[4, 60] Six different polymers were wet-spun to elucidate common trends (Figure 1c): Nylon 6, PCL, cellulose acetate, cellulose and two synthetic polypeptides produced within our group, namely PBLG and a recombinant spider silk protein eADF4(C16) (Figure 1c). The range of different polymer fibers were chosen to determine the impact of β-sheet introduction on their mechanical properties. It is important to note that cellulose fibers are formed from the deacetylation of already spun cellulose acetate fibers. Preliminary testing was performed using the Nylon 6 fiber to elucidate a target loading of Val NCA. Based on mechanical testing, loading the fibers with a 4:1 ratio of polymer to NCA (20 % NCA of the total solid) achieved the most interesting results to continue the study (Figure S1) and this loading remained consistent for each polymer. Preliminary testing was also performed on processing parameters such that consistent fiber spinning conditions could be achieved for each polymer system both in the absence and presence of Val NCA (Table 1)..

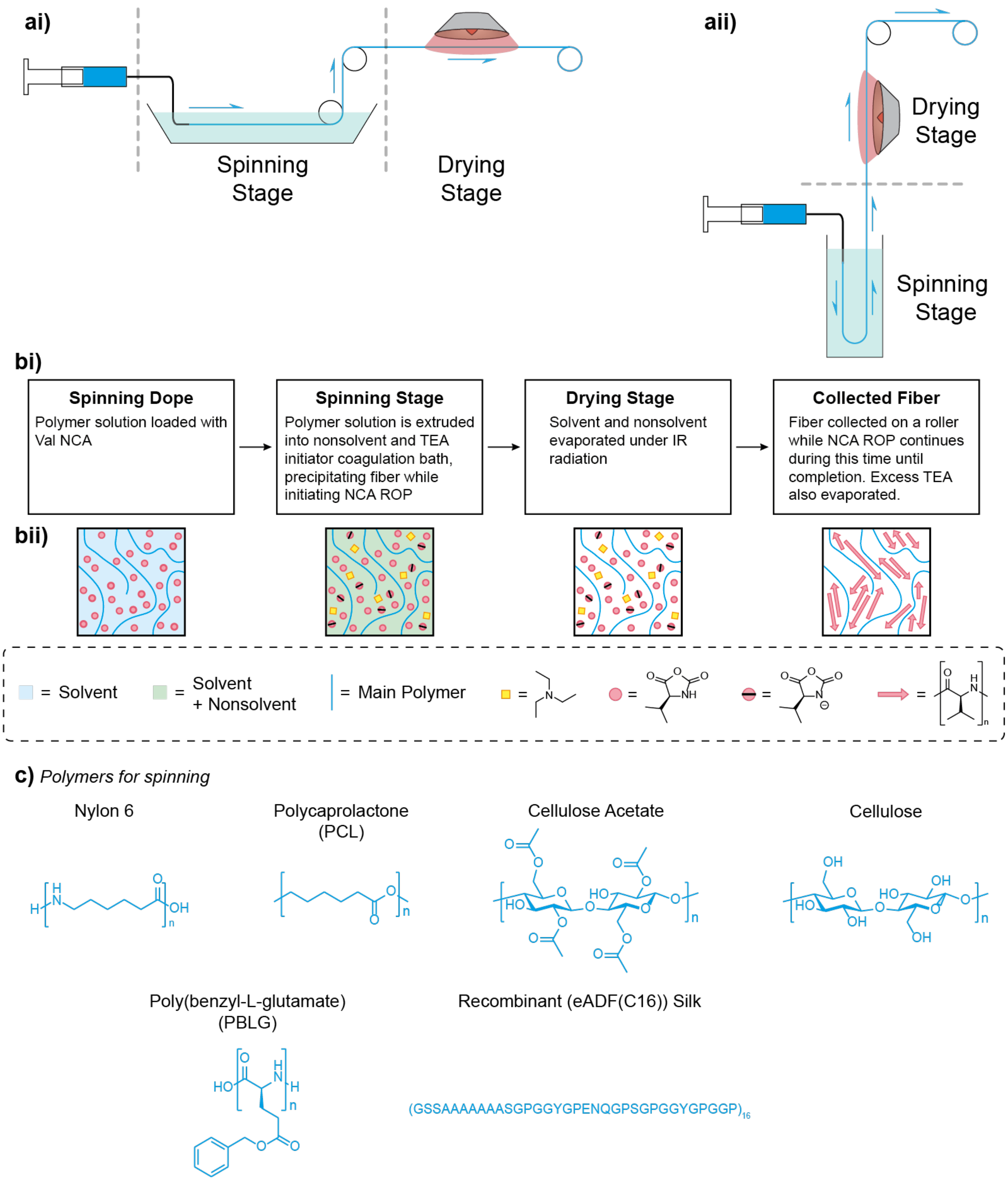

Figure 1: a) Schematic of the fiber wet spinning apparatus in i) horizontal and ii) vertical configurations. Vertical configurations were employed when the solvent was significantly denser than the nonsolvent. bi) Block flow diagram of stages of fiber fabrication and ii) the corresponding chemical schematic within each fiber during each stage. c) Chemical structure of all spun polymers.

Table 1: Summary of processing conditions for wet-spinning of polymers both in the absence and presence of Val NCA

Polymer[a)]	Spinning Dope Concentration [% w/v]	Spinning Dope Solvent[b)]	Coagulation Bath (with 2 % v/v TEA)[c)]	Configuration	Extrusion Rate [µL/min]
Nylon 6	30	80% v/v MeOH<br>20% v/v DMF	Water	Vertical	30
PCL	15	DMF	Water	Horizontal	45
Cellulose Acetate	15	DMF	Water	Horizontal	35
PBLG	10	DMF	Water	Horizontal	30
eADF4(C16)	30	90% v/v MeOH<br>10% v/v DCM	30% v/v Water<br>30% v/v Diethyl Ether<br>40% v/v Isopropanol	Vertical	30

[a)] PCL = "poly(caprolactone)"; PBLG = "poly(benzyl-L-glutamate)"; eADF4(C16) = recombinant spider silk protein

[b)] MeOH = "formic acid"; DMF = "dimethylformamide"; DCM = "dichloromethane"

[c)] TEA = "triethylamine"

Fourier transform infrared (FTIR) spectroscopy was used to analyze the Amide I band (1600 – 1700 cm^{-1}) to confirm both the introduction of polypeptides and their β-sheet conformation (Figure S2, Figure 2).[64] For fibers derived from non-polyamides, a clear peak was observed at 1633-1635 cm^{-1}, which specifically correlates to antiparallel β-sheet formation (Figure 2b-d).[65] The PBLG fibers had a peak correlating to α-helical conformation at 1652 cm^{-1},[66] while the introduction of PVal yielded a shoulder indicating antiparallel β-sheet structures, as with the non-polyamide polymers (Figure 2e). For Nylon 6, however, the peak indicating β-sheet formation did not appear as a distinct shoulder from the close neighboring peak already present without PVal at 1638 cm^{-1} (Figure 2a). Similarly, eADF4(C16) fibers did not show an easily distinguishable shoulder with a prominent peak at 1625 cm^{-1} due to the intrinsic β-sheet structure (Figure 2f).[67] Fourier self-deconvolution (FSD) of the Amide I band failed to determine a separate peak at 1633 cm^{-1}, necessitating an alternative analysis method.

In each case, the relative intensity of the prominent β-sheet peak compared to other peaks was determined (Table S1, Supporting Information). Here for each polymer, either the appearance or increase in relative signal of the β-sheet peak was determined upon the introduction of PVal. In the case of Nylon 6, the relative Amide I signal was compared to the CH and CH_2 aliphatic stretch at 2850 – 2950 cm^{-1} showing a distinct increase, attributed to an increase in the amide structure of β-sheets. For the two polypeptide-based fibers, FSD could be restricted to the

Amide I band as this conveniently gave a secondary structure content breakdown. For PBLG, the β-sheet content rose from 2 % to 11 %, while in the case of eADF4(C16) β-sheet content was found to increase from 46 % to 54 %, which is attributed to PVal. For Nylon 6 and eADF4(C16), this shift of the β-sheet band to preexisting amide I peaks may be due to numerous factors with one likely reason being the incorporation of β-sheets into the polymer's natural substructures.

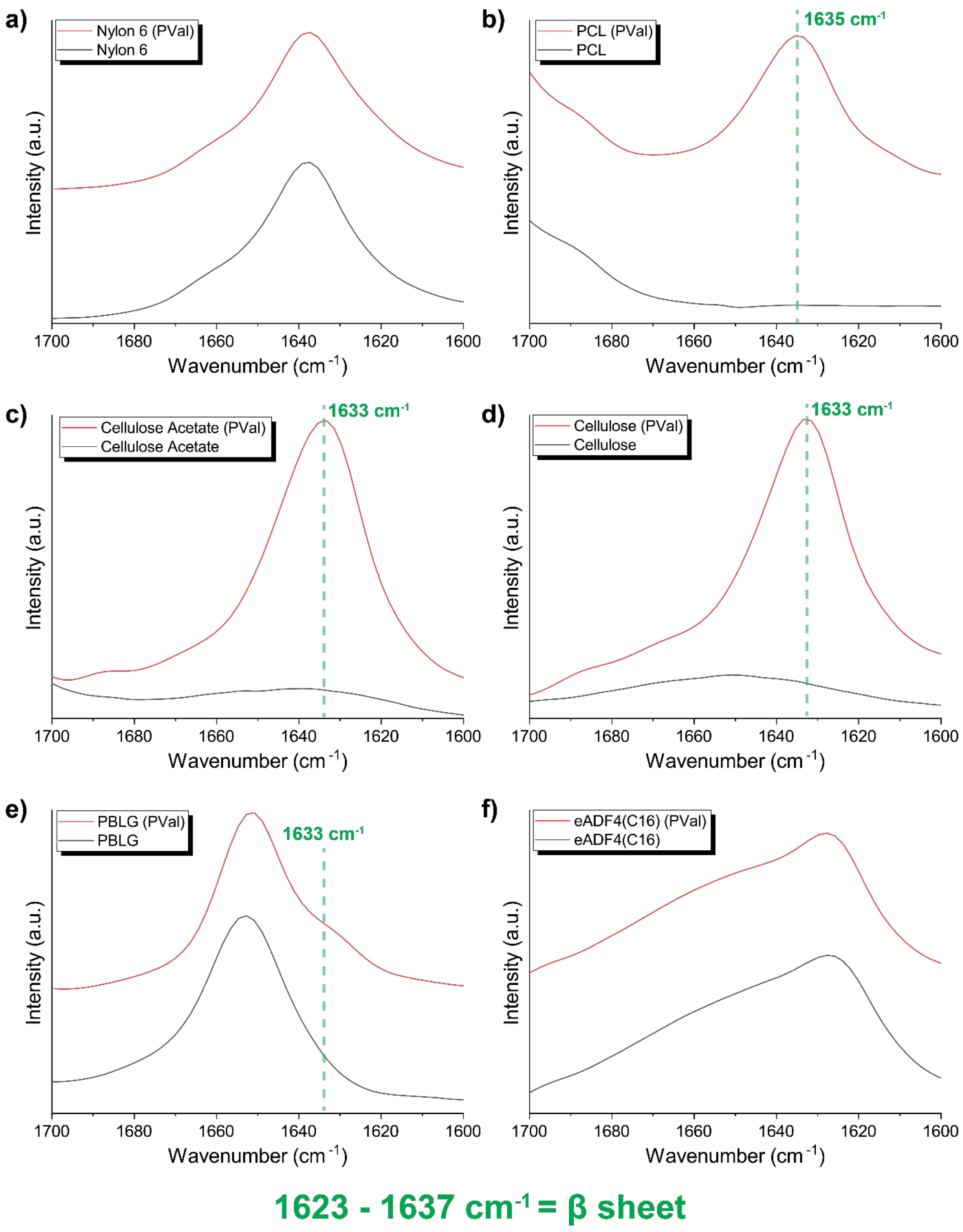

Figure 2: Fourier transform infrared (FTIR) spectra of the Amide I band (1600-1700 cm^{-1}) of fiber samples in the absence and presence of PVal for a) Nylon 6, b) poly(caprolactone) (PCL), c) cellulose acetate, d) cellulose, e) poly(benzyl-L-glutamate) (PBLG) and f) eADF4(C16) (recombinant spider silk).

Changes in both cross-sectional and surface morphology due to PVal introduction were visualized using scanning electron microscopy (SEM). Broadly, the overall roughness of the surface of all fibers increased upon introduction of PVal (Figure 3). This is potentially attributed to three different factors: i) the precipitation of the Val NCA disrupting the precipitation of the primary polymer matrix, ii) the conformational changes after aggregation and folding of the Val NCA and iii) the production of carbon dioxide during NCA ROP. The first of these points is exemplified in the overall fiber structure, where samples without any PVal were found to have surface features which are roughly parallel with the fiber axis (Figure 3i), while those containing PVal were found to twist to some degree (Figure 3iii). Nylon 6 samples were all found to have an undulated surface consistent with previous studies, but upon introduction of PVal (Figure 3aiii-iv), regions with greatly increased roughness were present on the surface of the fiber, consistent with β-sheet aggregates close to the surface. In contrast, PCL fibers were found to have comparatively little change in morphology despite being somewhat chemically similar to Nylon 6 (both are linear synthetic polymers with no pendant groups), with fibers being extremely porous both with and without β-sheet introduction (Figure 3b). A drastic decrease in homogeneity could be observed on the surface of cellulose acetate fibers with βsheet introduction and jagged ridges and edges appearing along the surface at a greater frequency, which is likely due to the combination of the aforementioned three factors (Figure 3c). This morphological difference between β-sheet loaded samples and their control counterpart was absent in cellulose fibers which were already porous without the introduction of PVal. The lack of change can be attributed to molecular rearrangement upon deacetylation, which then alters rearrangement after β-sheets have been formed, as opposed to during molecular rearrangement as with other fibers (Figure 3d). Interestingly, the two polypeptidebased fibers both resulted in clear morphological changes though with different impacts. In both cases, aggregation was not found to show any distinct visual difference from the rest of the polymer matrix. Striations on the PBLG fibers were still present, although non-directional bumps were also found across the surface (Figure 3e). Control eADF4(C16) fibers were found to have globule-like aggregates on the surface, but upon introduction of PVal the surfaces were a lot smoother (Figure 3f).

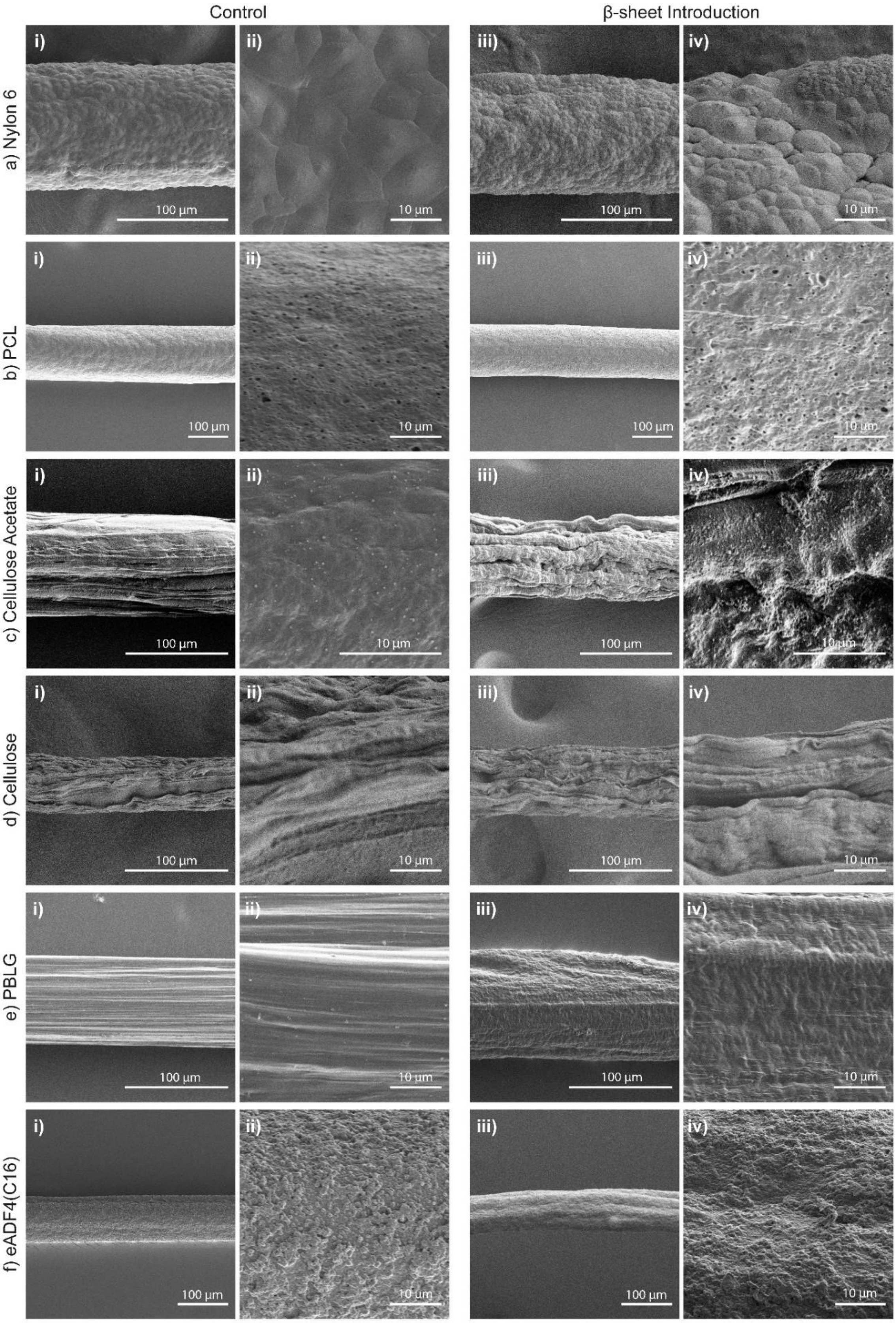

Figure 3: Scanning electron microscopy (SEM) images of fiber surfaces at i, iii) low magnification and ii, iv) high magnification of fiber samples i, ii) in the absence of and iii, iv) in the presence of PVal for a) nylon 6, b) poly(caprolactone) (PCL), c) cellulose acetate, d) cellulose, e) poly(benzyl-L-glutamate) (PBLG) and f) eADF4(C16).

Cross-sectional visualization of fibers further demonstrated the impact of introducing β-sheet forming polypeptides within the materials (Figure S3, Supporting Information). Interestingly, distinct regions of aggregation were only clearly visible in commercially available synthetic polymers (*i.e.,* those with long chain aliphatic backbones). In the case of Nylon 6, these distinct regions required close inspection, as regions were less than 2 μm in scale, while these regions in PCL samples were found to be more continuous at a larger scale (as highlighted in yellow). Furthermore, PCL fibers both had hollow interiors as a byproduct of fast coagulation. Due to their long aliphatic backbones, repetitive regions between hydrogen bonding groups are molecularly distant, hence resulting in the discrete regions where PVal chains favor hydrogen bonding with solely PVal in these two fibers. In contrast, all other fibers displayed a more homogeneous and continuous integration of β-sheets. Cellulose acetate fibers displayed much larger pores compared to Nylon 6 fibers due to processing. The introduction of PVal did appear to be homogeneously distributed clearly showing aggregates on a micrometer scale. The internal porous structure changed during the deacetylation process and the formation of cellulose fibers, though it was hard to discern any obvious changes in aggregation at a micrometer scale. The two polypeptide fibers did not possess the same macroporous nature as observed in the other fibers. PBLG fibers did not show discernable internal differences upon introduction of PVal. However, eADF(C16) fibers without PVal were found to possess a corona (highlighted in a yellow box in Figure S3fii)) surrounding the core, which was no longer present once PVal was included (highlighted in a yellow box in Figure S3fiv)), implying reduced microphase separation during spinning. While it could be confirmed that the introduction of PVal had introduced β-sheets within all fibers and presented microscale morphological changes, a further analysis of mesoscale structures was required to determine the molecular influence of β-sheet introduction.

To get a deeper understanding of the influence of the PVal β-sheets on the nanostructure, further analysis was performed using X-ray diffraction (XRD) and small-angle X-ray scattering (SAXS) on fiber bundles. According to literature, the interstrand spacing between β-strands leads to an interatomic spacing of d = 4.6-4.7 Å and intersheet spacing of ~10 Å between βsheet nanocrystals, cumulating in a cross-β structure indicative of antiparallel β-sheet nanocrystals.[28] X-ray diffraction (XRD) revealed a peak indicative of a d-spacing of 10 Å, within all samples upon introduction of PVal with the exception of eADF4(C16) (Figure 4). In accordance with literature, this peak was absent within Nylon 6,[29] PCL,[68] cellulose[31] and PBLG[67] without PVal, supporting the assumption that the d-spacing of 10 Å resulted from antiparallel β-sheets.

In case of cellulose acetate, a broad range of small crystallites was expected to lead to a broad peak around the same region (2θ ≈ 8°) without the addition of PVal,[33] but this peak became more defined and slightly shifted upon introduction of PVal indicating β-sheet intersheet spacing. Consistent with previous reports of β-sheet structures,[34] a *d*-spacing of 4.6 Å was observed as a slight shoulder in most instances, but most prominently in cellulose acetate and PCL. eADF4(C16) was again an exception in this case. In congruence with FTIR experiments, no distinct differences were observed between eADF4(C16) fibers with and without PVal. For both, in XRD a peak was observed at d = 4.4 Å (2θ = 20.1°) implying the presence of alanine-based β-sheet packing, which could be ascribed to the reduced side chain size.

The introduction of two distinct peaks, at 4.1 and 3.7 Å for cellulose acetate and PCL fibers upon modification was attributed to optimal (aliphatic) chain packing. Flexible linear polymers can form crystallites due to chain folding and subsequent formation of lamella or ideal packing of neighboring molecules. For cellulose acetate fibers, a *d*-spacing of 3.7 Å has previously been assigned to lattices structures of cellulose and its derivatives,[35] though they have also been assigned to natural β-sheets and thus cannot be specifically assigned to either component.

However, the peak at 2θ = 21.6 °, d = 4.1 Å has been reported for crystalline cellulose.[36] In the case of PCL, these peaks have been assigned as characteristic crystalline peaks of the polymer itself,[69] which was further defined upon introduction of PVal. This may be explained by the β-branched carbon of the PVal side chain which can bridge with the carbonyl oxygens and amide hydrogens of the polymers upon insertion.

Thus, differential scanning calorimetry (DSC) was used to determine if this correlated with a change in bulk crystallinity of PCL itself (Figure S4, Supporting Information). Upon introduction of β-sheets, fiber crystallinity was increased from 40% to 47% (7% increase in crystallinity). This contrasted with the study of Matolyak *et al*. who have previously shown a reduction in polyethylene glycol (PEG) crystallinity with increased β-sheet content in peptidepolyurea hybrid films, due to disruption of the crystalline structures within the polymer matrix (by intercalation in pre-existing crystalline regions).[45] It should be noted that matrix polymer crystallinity was reduced in their work in all samples compared to the control without peptide, due to the hydrogen bonding of peptidic units. We believe that the different results between their and our work results from the utilization of different material classes and the subsequent different fabrication techniques.

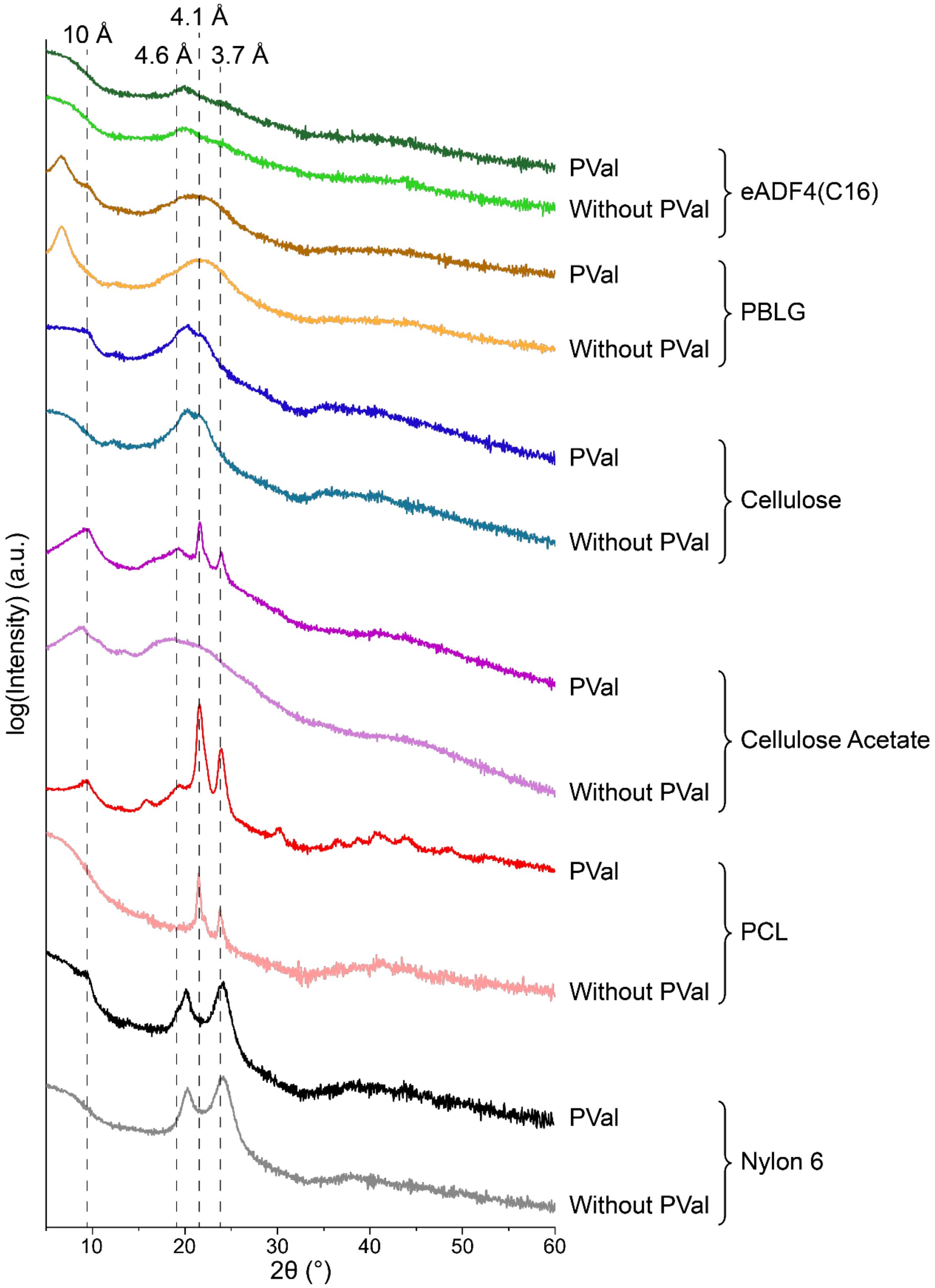

Figure 4: X-ray diffraction (XRD) spectra of fiber samples in the absence and presence of PVal made of Nylon 6, poly(caprolactone) (PCL), cellulose acetate, cellulose, poly(benzyl-Lglutamate) (PBLG) and eADF4(C16), respectively.

As the structure of a matter changes over different size regimes, small angle x-ray scattering (SAXS) (Figure S5), in combination with scaling law analysis (Equation 1, Table 2), was

performed to get an idea about the dimensionality of the (sub-) particle domains. Corresponding special scattering contributions (*I(q)*) characteristically scale with q^{-1}, q^{-2} and q^{-3} for 1D, 2D and 3D, respectively, while q^{-4} corresponds to a sharp interface or a homogeneous structure (Porod's law), whereas a lower exponent points to a diffuse interface or heterogeneous structure.[70] Nylon 6, PCL and cellulose acetate fibers exhibited mainly a q^{-4} dependence, both in the absence and presence of PVal. Note that shoulders which appear to correlate to crystallites of size of ~7 Å for Nylon and ~12 Å for PCL fibers appeared unaffected upon addition of PVal. In contrast, the scattering patterns of cellulose, PBLG and eADF4(C16) fibers changed upon addition of PVal, leading to the assumption that these materials become more porous/inhomogeneous due to PVal. Cellulose and PBLG fibers both exhibited a shift to n ~ 3.5 and ~ 3.3, respectively, indicating a partial increase in order within the bulk polymer matrix as caused by PVal introduction and intercalation of β-sheets. The q^{-4}/q^{-3} cross over point of PBLG with PVal suggested a correlation length of $\varepsilon \approx$ 75 Å (according to $2\pi/q = \varepsilon$). A possible explanation may be that long-range order of assemblies of PBLG α-helices became spatially separated by intercalating PVal chains. Notably, in case of eADF4(C16), which is a recombinant spider silk with inherent β-sheet structure, the correlation length decreased from $\varepsilon \approx$ 80 Å to $\varepsilon \approx$ 40 Å upon the addition of PVal, suggesting sheet fragmentation and consequently strain weakening due to crosslinks inside the molecular network.

Table 2: Summary of the mass fractal dimensions of all polymers in the absence and presence of PVal.

Polymer	Power law exponent (n)	
	Without PVal	With PVal
Nylon 6	4.0	4.0
PCL	4.1	4.0
Cellulose Acetate	3.9	3.9
Cellulose[a)]	4.0	3.5
PBLG	3.9	3.3
eADF4(C16)	3.5 ($q < 0.01$ Å^{-1})<br>2.2 ($q > 0.01$ Å^{-1})	3.9 ($q < 0.01$ Å^{-1})<br>2.0 ($q > 0.01$ Å^{-1})

As would be expected with the wide array of morphological changes exhibited over the range of tested polymers, the impact on mechanical properties was variable (Figure 5, Table S2, Supporting Information). Almost all fibers experienced an increased Young's modulus, which could be correlated to the presence of more rigid structures (Figure 5g). Notably, only PCL and cellulose acetate fibers, which were the two polymers which showed evidence of internal polymer crystallization, experienced a significantly increased strength upon PVal introduction (outlined in green), with an increase from 614 ± 63 to 1.34 ± 0.194 MPa (2.2 times increase) and 36.1 ± 5.2 to 154.4 ± 13.4 MPa (4.3 times increase) for PCL and cellulose acetate fibers, respectively (Figure 5b, c). Only Nylon 6 and PBLG fibers exhibited increases in elongation at break from 31.8 ± 2.2 % to 90.9 ± 5.9 % (2.9 times increase) and 87.8 ± 8.3 % to 159.2 ± 29 % (1.8 times increase), respectively (Figure 5a, d, outlined in blue). While the polymer matrix may have not been greatly affected in both cases, the strong hydrogen bonding between their polyamide backbones still allowed for energy dispersion throughout the fiber during tensile load. This potential for high degrees of hydrogen bonding further extended to both cellulose and eADF4(C16) fibers (outlined in red), however, both had undergone fundamental structural changes that counteracted this potential. The potential loss of nanocrystalline structure due to structural disruption in cellulose fibers would have contributed to reduced mechanical properties, but the increase in nanoscale order counteracted this to yield fibers with no statistically significant differences in mechanical properties. eADF4(C16) was the only polymer fiber to result in reduced mechanical capabilities in all assessments including Young's modulus (Figure 5f). Due to the design of eADF4(C16) leading to precise self-assembly characteristics, the disruption of the polymer matrix with PVal was expected to have a much more pronounced effect compared to other polymers resulting in the structural and consequential mechanical changes as described. The highly specific nature of spinning precisely designed proteins can be even further illustrated when comparing eADF4(C16) wetspun fibers in this work without PVal (35.5 ± 2.07 in tensile strength and 0.0753 ± 0.0051 mm/mm extension at break) to different variants of the eADF4 recombinant spider silk with specifically designed terminal domain. When such variants were spun using a specifically designed microfluidic multichannel device, superior mechanical properties were achieved (up to 834 MPa in tensile strength and 0.32 mm/mm extension at break), highlighting the precise self-assembly characteristics for this class of polymer.[7]

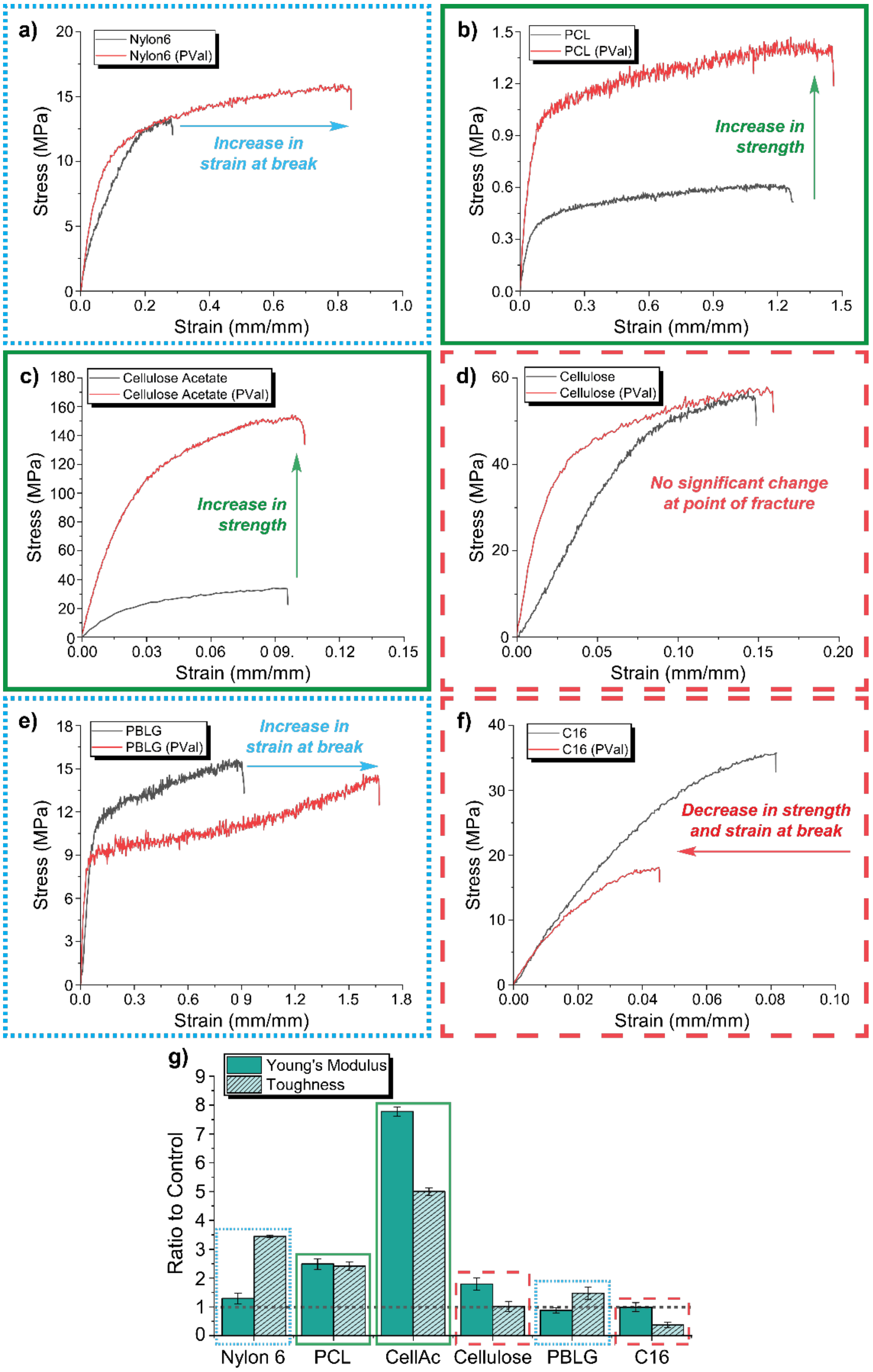

Figure 5: Representative tensile mechanical testing curves for fibers in the absence and presence of PVal made of a) Nylon 6, b) poly(caprolactone) (PCL), c) of cellulose acetate, d) cellulose, e) poly(benzyl-L-glutamate) (PBLG) and f) eADF4(C16). g) Summary of relative impact of PVal on strength, extension at break, Young's modulus and toughness for all polymers. All error bars represent standard deviation ($n = 5$). Samples experiencing a strength increase are outlined in green, samples experiencing an increase in strain at break are outlined in dashed

blue and samples experiencing no significant difference or reduced mechanical properties are outlined in red.

We hypothesize that the varying effects on mechanical properties of the fibers have been correlating with the internal crystalline structure of the polymer (Figure 6). Previous works have shown the introduction of secondary structures into a composite system affecting the natural organization of amorphous polymers, with β-sheet structures specifically showing potential to alter existing polymer crystal structures.[45] In the case of recombinant spider silk (eADF4(C16)), which shows intrinsic β-sheet crystalline structure,[71] the hypothesis suggested that such structures are disrupted by intercalating PVal β-sheets, thus resulting in inferior mechanical properties (Figure 6a). This is supported by the reduction in chain organization at a nanoscale upon β-sheet introduction as shown in SAXS measurements. Increased inhomogeneity was also observed in cellulose fibers, but they also experienced an increase in fractal dimensional order, indicating that ordered structures were maintained (Figure 6b). The net result yielded insignificant mechanical property changes. In contrast, Nylon 6, PBLG, PCL and cellulose acetate fibers all experienced significant mechanical property alterations, but different in nature. In this context, it is hypothesized that Nylon 6 and PBLG polymer chains are able to undergo significant intermolecular bonding with PVal domains,[72, 73] thus resulting in a nanocomposite with increased crystallinity and thus increased tensile strain at break (Figure 6c). This is supported by the prevalence of intersheet spacing within β-sheet nanocrystals identified in the XRD spectra. It is further interesting to contrast the instance of PBLG, which primarily contains α-helices in its structure, against the case of eADF4(C16) which contains βsheets. Unlike fibers fabricated from eADF4(C16), no adverse loss in structure is identified in SAXS measurements upon PVal introduction. However, PCL and cellulose acetate fibers both exhibited an increase in tensile strength due to increased bulk crystallinity within the polymer, which was induced by β-sheet crystals (Figure 6d). This increase was evidenced in the case of PCL through DSC measurements and the presence of cellulose acetate lattice structures only present in samples containing PVal. The difference between the latter two sets of polymeric fibers may be due to the formation of a low density of hydrogen bond acceptors in PCL and cellulose acetate compared to the other polymers, but this is speculative at best. Nonetheless, structural data supported this hypothesis linking different mechanical property alterations to specific changes in ordered structure.

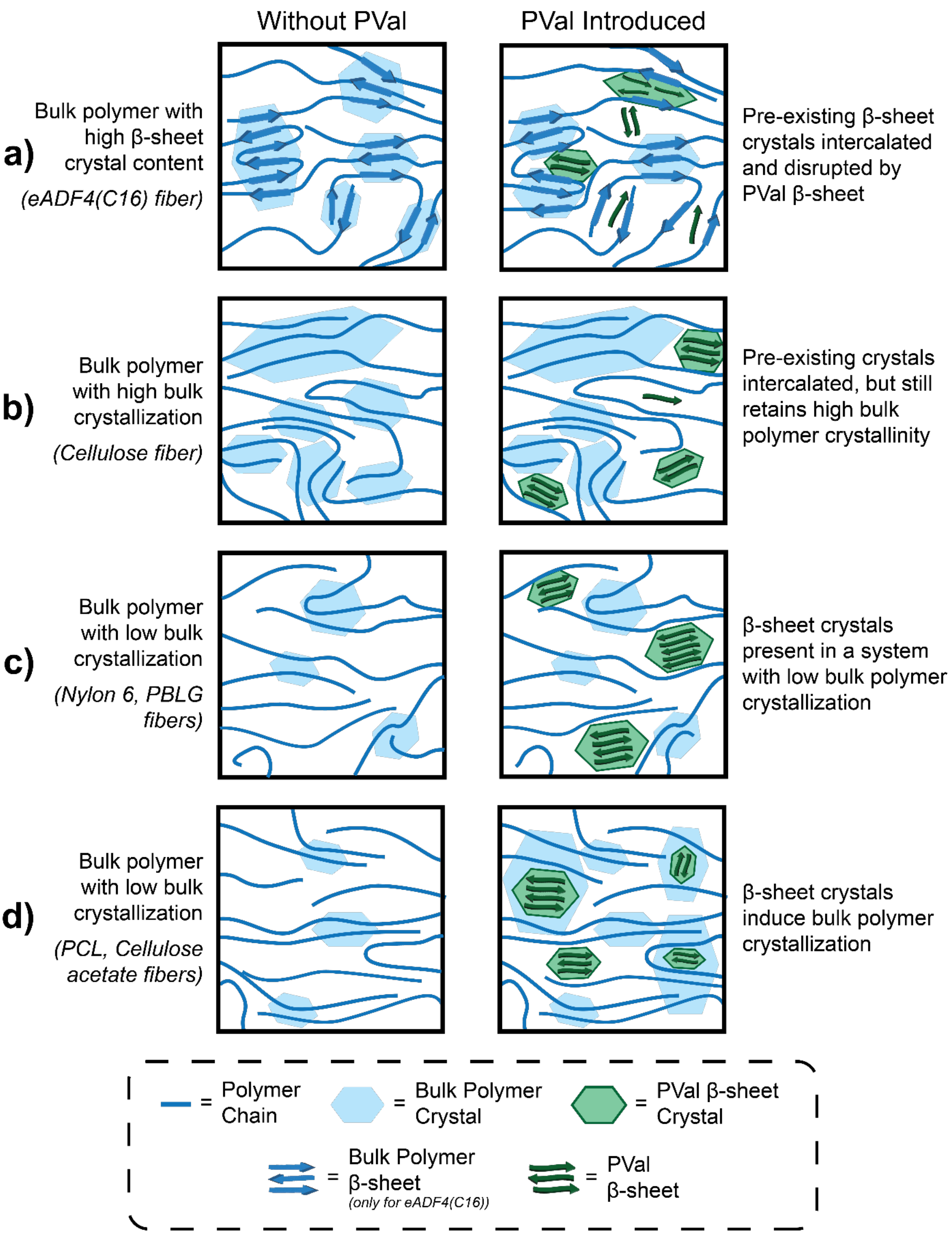

Figure 6: Model for impact of PVal-induced β-sheet crystals on different polymer systems based on interactions with bulk polymer crystallization. a) In polymer systems already containing β-sheet crystals (i.e. eADF4(C16) fibers), these crystals are disrupted. b) In polymer systems with high bulk crystallinity (i.e. cellulose fibers), the overall degree of crystallinity is not significantly altered. In polymer systems with low crystallinity, the introduction of PValinduced β-sheet crystals increases crystallinity either c) directly due to the β-sheet crystals themselves

(for Nylon 6 and PBLG fibers) or d) through bulk polymer crystallization (for PCL and cellulose acetate fibers).

3. Conclusion

In summary, we have developed a novel and facile methodology for introducing β-sheets into a range of different polymer fibers and investigated the subsequent changes in morphology due to this technique. By introducing Val NCA monomer into the spinning dope, polymerization could be initiated during the fabrication of different synthetic, polysaccharide, and polypeptide fibers. Upon investigation into substructures, cross-β structures, typical of β-sheet inclusion, were found to be present in the majority of fibers. However, further intermolecular interactions between the subsequent PVal-based β-sheets and the polymers increased the bulk crystallization in PCL and cellulose acetate fibers, which both have a low density of hydrogen bond acceptors compared to the other polymers. Such fibers were found to have increased tensile strength upon β-sheet introduction of up to 4.3 times. Other polymers which did not exhibit a loss in structure – namely Nylon 6 and PBLG – exhibited an increase in extensibility at break of up to 2.9 times due to the increased density of hydrogen bonding. However, this increased hydrogen bonding was found to disrupt the internal structure of other polymers more significantly. In the case of cellulose, no significant mechanical change was observed, where a loss of nanoscale structure was found to be counterbalanced by an overall increase in nanoscale structural order. In the case of the recombinant spider silk (eADF4(C16)), the reduced nanoscale structural order resulted in all mechanical properties being significantly inferior to the counterpart without PVal.

Thus, we have established a novel and facile fiber fabrication system to introduce β-sheet structures without complex or multi-step reactions, garnering significant changes in fiber properties and thus guide the future development of tough materials and fibers.

4. Materials and Methodology

Materials: Acetone (tech. grade, VWR chemicals), alginic acid sodium salt (300-350 kDa, Carl Roth), *N,N*-dimethyl formamide (DMF, 99.8%, anhydrous, Sigma-Aldrich), diethyl ether (Et_2O, AR grade, Chem-Supply), dichloromethane (DCM, AR grade, Chem-Supply), cellulose acetate (MW 100,000, Arcos Chemicals), ethanol (tech. grade, VWR chemicals), formic acid (puriss. p.a. ≥98%, Sigma Aldrich), H-L-valine-OH (>99%, Mimotopes), H-L-Glu(OBzl)-OH (>99%, Mimotopes), magnesium sulfate (anh. $MgSO_4$, ≥97%, Merck), α-pinene (98%, Merck), sodium hydrogen carbonate ($NaHCO_3$, AR grade, Chem-Supply), sodium hydroxide (NaOH; ≥97%,

Merck), triethylamine (TEA, ≥99.5%, Sigma-Aldrich), triphosgene (≥98%, Merck), and Nylon 6 (Sigma Aldrich) were all used as received without further purification. Saturated brine solution was prepared from sodium chloride (NaCl, AR grade, Chem-Supply) in DI water. Tetrahydrofuran (anh. THF, inhibitor free, >99.9%, Merck), ethyl acetate (anh. EtOAc, >99.9% Honeywell) and were purified by passage through a solvent purification system (SBPT-1, LC Technologies, USA) containing 4Å sieves under Argon gas. Hexane (AR, Chem-Supply) was distilled over calcium hydride under nitrogen prior to use.

Synthesis of Valine NCA (Val NCA) and Benzyl-protected Glutamic Acid (Glu(OBzl) NCA): Both NCAs were synthesized using modified versions of previous reported methods.[60] In a typical experiment Val (10.0 g, 42.2 mmol) was added to a 500 mL three-necked round bottom flask and suspended in 200 mL of anh. THF with α-pinene (31.1 mL, 96.9 mmol, 1.15 excess moles) under nitrogen. After heating to 60 ˚C, triphosgene (10.1 g, 16.9 mmol, 1.2 excess moles) was added and the mixture was stirred at for 2 h. Solvent was then removed from the clear solution under reduced pressure and replaced with 100 mL of anh. EtOAc. The solution was cooled to -18 ˚C before washing with sat. brine (1 × 100 mL) and then 5 w/v% aq. $NaHCO_3$ (1 × 100 mL), with the subsequent organic phase dried over $MgSO_4$. The filtered solution was reduced to dryness *in vacuo* and recrystallized using anh. hexane at -18 ˚C over 16 h. The solids were then filtered and redissolved into anh. EtOAc before being precipitated into anh. hexane. The precipitate was then dried *in vacuo* over 24 h to afford a colorless solid and stored under argon at -80 ˚C.

Val NCA (10.2 g, 83% yield): ^{1}H NMR (400 MHz, $CDCl_3$, δ): 1.00-1.09 (m, J = 7.0 Hz, 3H; CH-(C**H**$_3$)$_2$), 2.27 (1, 1H; -C**H**-(CH$_3$)$_2$), 4.24 (d, J = 4.4 Hz, 1H; -C**H**-NH-), 6.79 (s, 1H; ring N**H**)

Glu(OBzl) NCA (9.7 g, 87 % yield): ^{1}H NMR (400 MHz, $CDCl_3$, δ): 2.08−2.31 (m, 2H; -C**H**$_2$CH$_2$-CO-), 2.55 (t, J = 6.8 Hz, 2H; -CH$_2$-C**H**$_2$-CO-), 4.42 (t, J = 6.0 Hz, 1H; -C**H**-NH-), 5.11
(s, 2H; -C**H**$_2$-ArH), 6.47 (s, 1H; ring N**H**), 7.35−7.40 (m, 5H; Ar**H**).

Synthesis of Poly(benzyl-L-gluatamic acid) (PBLG): Glu(OBzl) NCA (1.50 g) was dissolved in 50 mL of anh. DCM under nitrogen in a 100 mL RBF. To the stirring solution was added TEA, and the clear solution was stirred under an argon bleed at room temperature for 24 h. The

reaction mixture was then precipitated into and subsequently rinsed with Et_2O which was then dried and stored under vacuum to afford a clear tacky solid as the final product (2.84 g, 78.5% yield). ^{1}H NMR (400 MHz, DMSO-d_6, δ): 2.04−2.48 (m, 2H; -C**H2**-C**H2**-CO-), 3.90 (t, 1H; C**H**-N-CO-), 5.00 (s, 2H, -C**H2**-ArH), 5.59 (s, 1H; ring N**H**), 7.19−7.30 (m, 5H; Ar**H**), Average M_W = 333 kDa, PDI = 1.21.

Production of eADF4(C16) Recombinant Protein: Genetically modified recombinant spider silk protein eADF4(C16) was produced as previously reported.[74] eADF4(C16) was expressed in *Escherichia coli* (BL21 gold) and cells were grown in a fermenter using a fed batch system.[74] The product was purified using a heat step and ammonium sulfate precipitation as described previously.[74]

Wet Spinning of Polymeric Fibers: Polymer solutions with assigned spinning dope concentrations were dissolved over 4 h (until complete dissolution in anhydrous spinning dope solvent under nitrogen according to Table 1. For samples loaded with NCA, NCA was then introduced into the solution at a 4:1 ratio of polymer to NCA. The subsequent solution was extruded into the coagulation bath with 2% v/v TEA as an initiator at a continuous rate. Upon precipitation, the fiber was drawn out of the coagulation bath and dried under an infrared lamp before being collected. After 24 h, the collected fibers were washed in the coagulation bath solution without TEA, 3 times and subsequently dried under an air stream for 48 h.

Deacetylation of Cellulose Acetate Fibers: Cellulose acetate fibers were soaked in a solution of 0.5 N aq. 50% EtOH / 50% H_2O for 24 h. Subsequent cellulose fibers were washed progressively in 50% EtOH / 50% H_2O, 25% EtOH / 75% H_2O solution and H_2O before subsequent drying under an air stream for 48 h.

Mechanical Testing: Tensile mechanical testing was performed using an ElectroForce 3200 (TA Instruments, DE, USA) with 2.5 N load cells. Small sections of fibers were randomly selected and fixed onto plastic frames with a gap of 3 mm using two-component glue in case of dry samples. The glue was left to dry under a fume hood for at least 24 h. The frames were then fixed between the clamps of the testing device and pulled apart until failure at a rate of 0.01 mm s^{-1} at a relative humidity of 65%, and room temperature. The Young's modulus was obtained by calculating the slope of the stress–strain curve in the linear elastic region. The fiber toughness

was calculated by integrating the stress–strain curve. The measurements were repeated for a minimum of five individual samples.

Equipment: ATR-FTIR spectroscopy was performed with a Bruker Hyperion 1000 microscope with a dedicated ATR-objective (Bruker Optics GmbH, Ettlingen, Germany). The ATR-crystal was brought into contact with the sample. The microscope was continuously purged with dry air, and the MCT detector was cooled with liquid nitrogen. The FTIR spectra were recorded with a resolution of 2 cm^{-1}. One hundred scans per measurement were performed. Three samples per layer with three spots per sample were measured. Scanning electron microscopy (SEM) was attained using a Leo 1530 Gemini (Zeiss, Oberkochen, Germany) at an accelerating voltage of 3 kV. Samples were sputtered with 1.3 nm platinum. SAXS was performed using the small-angle X-ray system "Double Ganesha AIR" (SAXSLAB, Denmark). A power law analysis was performed by fitting the curve to Equation 1 to subsequently determine internal structure.[75]

$$(q) \propto q^{-n} \quad (1)$$

Where I is the scattering intensity q
is the scanning vector size ($Å^{-1}$) n
is the mass fractal dimension

The X-ray source is a rotating anode (copper, MicroMax 007HF, Rigaku Corporation, Japan) providing a microfocused beam. XRD patterns were obtained on a Bragg–Brentanotype diffractometer (Empyrean, Malvern Panalytical BV, The Netherlands) equipped with a
PIXcel-1D detector using Cu Kα radiation (λ = 1.54187 Å). Differential scanning calorimetry (DSC) measurements on PCL were carried out on a PerkinElmer DSC 8500 with a heating rate of 40 °C min^{-1}. Subsequent crystallization was determined by determining the heat of melting of crystalline structures and determining it as a percentage of the heat of melting of 100 % crystalline PCL.[76]

Supporting Information

Supporting Information is available from the Wiley Online Library.

Acknowledgements

This work was performed in part at the Materials Characterization and Fabrication Platform (MCFP) at the University of Melbourne. N.J.C thanks The University of Melbourne for providing Australian Government Research Training Program Scholarship (AGRTP) for providing funds for travel. This work is supported by grants DFG SCHE603/ 23-1. N.J.C., S.L., G.G.Q. and T.S. acknowledge funding from the German Academic Exchange Service (DAAD) through its thematic network Bayreuth-Melbourne Colloid/Polymer Network sponsored from funds of the Federal Ministry of Education and Research (BMBF).

Conflict of Interest

T.S. is co-founder and shareholder of the company AMSilk GmbH.

Author Contributions

Nicholas J. Chan: Conceptualization; methodology; investigation; formal analysis; data curation; visualization; writing – original draft preparation; writing – review and editing Sarah Lentz: Conceptualization; investigation; formal analysis; writing – original draft preparation; writing – review and editing

Paul A. Gurr: Supervision; formal analysis; writing – review and editing

Shereen Tan: Supervision; writing – review and editing

Mona Schultebeyring: Investigation; formal analysis

Sabine Rosenfeldt: Investigation

Anna Schenk: Investigation

Thomas Scheibel: Conceptualization; funding acquisition; resources; supervision; writing – review and editing

Greg G. Qiao: Conceptualization; funding acquisition; resources; supervision; writing – review and editing

Data Availability Statement

The data that support the findings of this study are available from the corresponding author upon reasonable request.

Received: ((will be filled in by the editorial staff))

Revised: ((will be filled in by the editorial staff))

Published online: ((will be filled in by the editorial staff))

References

[1] Zhang, C.; Mcadams II, D. A.; Grunlan, J. C., *Adv. Mater.* **2016,** *28* (30), 6292-6321.

[2] Ganewatta, M. S.; Wang, Z.; Tang, C., *Nat. Rev. Chem.* **2021,** *5* (11), 753-772.

[3] Bonduelle, C., *Polym. Chem.* **2018,** *9* (13), 1517-1529.

[4] Fujiwara, K.; Toda, H.; Ikeguchi, M., *BMC Struct. Biol.* **2012,** *12* (1), 18.

[5] Mondal, S.; Jacoby, G.; Sawaya, M. R.; Arnon, Z. A.; Adler-Abramovich, L.; Rehak, P.; Vuković, L.; Shimon, L. J. W.; Král, P.; Beck, R.; Gazit, E., *J. Am. Chem. Soc.* **2019,** *141* (1), 363-369.

[6] Brooks, A. E.; Steinkraus, H. B.; Nelson, S. R.; Lewis, R. V., *Biomacromolecules* **2005,** *6* (6), 3095-3099.

[7] Saric, M.; Eisoldt, L.; Döring, V.; Scheibel, T., *Adv. Mater.* **2021,** *33* (9), 2006499. [8] Correa-Garhwal, S. M.; Clarke, T. H.; Janssen, M.; Crevecoeur, L.; McQuillan, B. N.; Simpson, A. H.; Vink, C. J.; Hayashi, C. Y., *Sci. Rep.* **2019,** *9* (1), 13656.

[9] Xu, M.; Lewis, R. V., *Proc. Natl. Acad. Sci. U.S.A.* **1990,** *87* (18), 7120-7124. [10] Plaza, G. R.; Pérez-Rigueiro, J.; Riekel, C.; Perea, G. B.; Agulló-Rueda, F.; Burghammer, M.; Guinea, G. V.; Elices, M., *Soft Matter* **2012,** *8* (22), 6015-6026.

[11] Grubb, D. T.; Jelinski, L. W., *Macromolecules* **1997,** *30* (10), 2860-2867.

[12] Riekel, C.; Bränden, C.; Craig, C.; Ferrero, C.; Heidelbach, F.; Müller, M., *Int. J. Biol. Macromol.* **1999,** *24* (2), 179-186.

[13] Hakimi, O.; Knight, D. P.; Vollrath, F.; Vadgama, P., *Compos. B Eng.* **2007,** *38* (3), 324-337.

[14] Johnson, J. C.; Korley, L. T. J., *Soft Matter* **2012,** *8* (45), 11431-11442.

[15] Chan, N. J.; Lentz, S.; Gurr, P. A.; Scheibel, T.; Qiao, G. G., *Prog. Polym. Sci.* **2022,** *130*, 101557.

[16] Khan, A. Q.; Shafiq, M.; Li, J.; Yu, K.; Liu, Z.; Zhou, X.; Zhu, M., *SmartMat* **2023,** *4* (6), e1189.

[17] Mondal, S.; Das, S.; Nandi, A. K., *Soft Matter* **2020,** *16* (6), 1404-1454.

[18] Murugesan, S.; Scheibel, T., *Adv. Funct. Mater.* **2020,** *30* (17), 1908101.

[19] Mondal, S., *Polym.-Plast. Technol. Mater.* **2018,** *57* (13), 1377-1391.

[20] Kumar, A.; Sharma, K.; Dixit, A. R., *J. Mater. Sci.* **2019,** *54* (8), 5992-6026.

[21] Qian, Y.; Di, S.; Wang, L.; Li, Z., *J. Mater. Chem. B* **2021,** *9* (33), 6521-6535.

[22] Chen, H.; Xu, J.; Sun, J.; Jiang, Y.; Zheng, W.; Hu, W.; Qian, H., *Asian J. Pharm. Sci.* **2024,** *19* (3), 100911.

[23] Wei, L.; Cai, C.; Lin, J.; Chen, T., *Biomaterials* **2009,** *30* (13), 2606-2613.

[24] Patel, M.; Nakaji-Hirabayashi, T.; Matsumura, K., *J. Biomed. Mater. Res. A* **2019,** *107* (5), 1094-1106.

[25] Zhao, B.; Zhang, X.; Bickle, M. S.; Fu, S.; Li, Q.; Zhang, F., *Nanoscale* **2024,** *16* (5), 2250-2264.

[26] Goh, S.; Platt, A.; Rutledge, K.; Lee, I., *J. Polym. Sci. Part A: Polym. Chem.* **2008,** *46* (16), 5381-5389.

[27] Wibowo, S. H.; Sulistio, A.; Wong, E. H. H.; Blencowe, A.; Qiao, G. G., *Aust. J. Chem.* **2014,** *67* (4), 598-602.
[28] Chan, N. J.; Lentz, S.; Gurr, P. A.; Tan, S.; Scheibel, T.; Qiao, G. G., *Angew. Chem. Int. Ed.* **2022,** *61* (9), e202112842.
[29] Gauthier, M. A.; Klok, H.-A., *Chem. Commun.* **2008,** (23), 2591-2611.
[30] Canalle, L. A.; Löwik, D. W. P. M.; van Hest, J. C. M., *Chem. Soc. Rev.* **2010,** *39* (1), 329-353.
[31] Shu, J. Y.; Panganiban, B.; Xu, T., *Annu. Rev. Phys. Chem.* **2013,** *64* (1), 631-657.
[32] Ibarboure, E.; Papon, E.; Rodríguez-Hernández, J., *Polymer* **2007,** *48* (13), 3717-3725.
[33] Moon, H. J.; Choi, B. G.; Park, M. H.; Joo, M. K.; Jeong, B., *Biomacromolecules* **2011,** *12* (4), 1234-1242.
[34] Maslovskis, A.; Guilbaud, J. B.; Grillo, I.; Hodson, N.; Miller, A. F.; Saiani, A., *Langmuir* **2014,** *30* (34), 10471-10480.
[35] Murphy, R.; Borase, T.; Payne, C.; O'Dwyer, J.; Cryan, S. A.; Heise, A., *RSC Adv.* **2016,** *6* (28), 23370-23376.
[36] Gudeangadi, P. G.; Uchida, K.; Tateishi, A.; Terada, K.; Masunaga, H.; Tsuchiya, K.; Miyakawa, H.; Numata, K., *Polym. Chem.* **2020,** *11* (30), 4920-4927.
[37] Guo, X.; Yang, B.; Chen, J., *Biomacromolecules* **2024,** *25* (6), 3642-3650.
[38] Lai, H.; Chen, X.; Lu, Q.; Bian, Z.; Tao, Y.; Wang, X., *Chem. Commun.* **2014,** *50* (91), 14183-14186.
[39] Huang, H.; Hu, J.; Zhu, Y., *Macromol. Biosci.* **2013,** *13* (2), 161-166.
[40] Matolyak, L. E.; Keum, J. K.; Van de Voorde, K. M.; Korley, L. T. J., *Org. Biomol. Chem.* **2017,** *15* (36), 7607-7617.
[41] Johnson, J. C.; Wanasekara, N. D.; Korley, L. T. J., *Biomacromolecules* **2012,** *13* (5), 1279-1286.
[42] Jang, D.; Thompson, C. B.; Chatterjee, S.; Korley, L. T. J., *Mol. Syst. Des. Eng.* **2021,** *6* (12), 1003-1015.
[43] Johnson, J. C.; Wanasekara, N. D.; Korley, L. T. J., *J. Mater. Chem. B* **2014,** *2* (17), 2554-2561.
[44] Jang, D.; Beckett, L. E.; Keum, J.; Korley, L. T. J., *J. Mater. Chem. B* **2023,** *11* (24), 5594-5606.
[45] Matolyak, L. E.; Thompson, C. B.; Li, B.; Keum, J. K.; Cowen, J. E.; Tomazin, R. S.; Korley, L. T. J., *Biomacromolecules* **2018,** *19* (8), 3445-3455.
[46] Choi, B. G.; Park, M. H.; Cho, S. H.; Joo, M. K.; Oh, H. J.; Kim, E. H.; Park, K.; Han, D. K.; Jeong, B., *Biomaterials* **2010,** *31* (35), 9266-72.
[47] Yun, E. J.; Yon, B.; Joo, M. K.; Jeong, B., *Biomacromolecules* **2012,** *13* (4), 1106-1111.
[48] Zhang, J.; Liu, Y.; Sun, J.; Gu, R.; Ma, C.; Liu, K., *Materials Today Adv.* **2020,** *8*, 100095.
[49] Haynl, C.; Hofmann, E.; Pawar, K.; Förster, S.; Scheibel, T., *Nano Lett.* **2016,** *16* (9), 5917-5922.
[50] Mu, B.; Hassan, F.; Yang, Y., *Green Chem.* **2020,** *22* (5), 1726-1734.
[51] Bier, M. C.; Kohn, S.; Stierand, A.; Grimmelsmann, N.; Homburg, S. V.; Rattenholl, A.; Ehrmann, A., *IOP Conference Series: Materials Science and Engineering* **2017,** *254*, 192004.
[52] Yan, J.; Zhou, G.; Knight, D. P.; Shao, Z.; Chen, X., *Biomacromolecules* **2010,** *11* (1), 1-5.

[53] Zhang, F.; Lu, Q.; Yue, X.; Zuo, B.; Qin, M.; Li, F.; Kaplan, D. L.; Zhang, X., *Acta Biomater.* **2015,** *12*, 139-145.
[54] Ling, S.; Qin, Z.; Li, C.; Huang, W.; Kaplan, D. L.; Buehler, M. J., *Nat. Commun.* **2017,** *8* (1), 1387.
[55] Heidebrecht, A.; Eisoldt, L.; Diehl, J.; Schmidt, A.; Geffers, M.; Lang, G.; Scheibel, T., *Adv. Mater.* **2015,** *27* (13), 2189-2194.
[56] Xia, X.-X.; Qian, Z.-G.; Ki, C. S.; Park, Y. H.; Kaplan, D. L.; Lee, S. Y., *Proc. Natl. Acad. Sci. U.S.A.* **2010,** *107* (32), 14059-14063.
[57] Rasines Mazo, A.; Allison-Logan, S.; Karimi, F.; Chan, N. J.-A.; Qiu, W.; Duan, W.; O'Brien-Simpson, N. M.; Qiao, G. G., *Chem. Soc. Rev.* **2020,** *49* (14), 4737-4834.
[58] Gu, L.; Jiang, Y.; Hu, J., *Adv. Mater.* **2019,** *31* (48), 1904311.
[59] Rathore, O.; Sogah, D. Y., *J. Am. Chem. Soc.* **2001,** *123* (22), 5231-5239.
[60] Chan, N. J.-A.; Gu, D.; Tan, S.; Fu, Q.; Pattison, T. G.; O'Connor, A. J.; Qiao, G. G., *Nat. Commun.* **2020,** *11* (1), 1630.
[61] Jacobs, J.; Pavlović, D.; Prydderch, H.; Moradi, M.-A.; Ibarboure, E.; Heuts, J. P. A.; Lecommandoux, S.; Heise, A., *J. Am. Chem. Soc.* **2019,** *141* (32), 12522-12526.
[62] Vacogne, C. D.; Schlaad, H., *Chem. Commun.* **2015,** *51* (86), 15645-15648.
[63] Zhao, W.; Gnanou, Y.; Hadjichristidis, N., *Chem. Commun.* **2015,** *51* (17), 3663-3666.
[64] Hofmaier, M.; Urban, B.; Lentz, S.; Borkner, C. B.; Scheibel, T.; Fery, A.; Müller, M., *J. Phys. Chem. B* **2021,** *125* (4), 1061-1071.
[65] Lomont, J. P.; Ostrander, J. S.; Ho, J.-J.; Petti, M. K.; Zanni, M. T., *J. Phys. Chem. B* **2017,** *121* (38), 8935-8945.
[66] Tsuboi, K.; Marcelletti, E.; Matsumoto, H.; Ashizawa, M.; Minagawa, M.; Furuya, H.; Tanioka, A.; Abe, A., *Polym. J.* **2012,** *44* (4), 360-365.
[67] Zou, Y.; Li, Y.; Hao, W.; Hu, X.; Ma, G., *J. Phys. Chem. B* **2013,** *117* (15), 4003-4013.
[68] Bojedla, S. S. R.; Chameettachal, S.; Yeleswarapu, S.; Nikzad, M.; Masood, S. H.; Pati, F., *J. Biomed. Mater. Res. A* **2022,** *110* (7), 1386-1400.
[69] Woo, H. J.; Majid, S. R.; Arof, A. K., *Solid State Ionics* **2013,** *252*, 102-108.
[70] Glatter, O.; Kratky, O.; Kratky, H., *Small angle X-ray scattering*. Academic press: **1982**.
[71] Metwalli, E.; Slotta, U.; Darko, C.; Roth, S. V.; Scheibel, T.; Papadakis, C. M., *Appl. Phys. A* **2007,** *89* (3), 655-661.
[72] Deshmane, S.; Hayashi, T.; Sederel, W.; Anderson, J. M., *Biopolymers* **1978,** *17* (12), 2851-2864.
[73] Chai, Z.; BelBruno, J. J., *J. Mol. Recognit.* **2015,** *28* (11), 651-655.
[74] Huemmerich, D.; Helsen, C. W.; Quedzuweit, S.; Oschmann, J.; Rudolph, R.; Scheibel, T., *Biochemistry* **2004,** *43* (42), 13604-13612.
[75] Johansen, D.; Trewhella, J.; Goldenberg, D. P., *Protein Sci.* **2011,** *20* (12), 1955-1970.
[76] Crescenzi, V.; Manzini, G.; Calzolari, G.; Borri, C., *Eur. Polym. J.* **1972,** *8* (3), 449-463.

Table of Contents: β-sheet nanocrystals were embedded into a range of different polymeric fibers by introducing a soluble monomer into the spinning dope solution and polymerizing after fiber formation. Polymer crystallization was found in specific polymeric fibers, resulting in an

increase in tensile strength 2.2 and 4.3 times for poly(caprolactone) and cellulose acetate respectively.

N. J. Chan, S. Lentz, P. A. Gurr, S. Tan, M. Schultebeyring, S. Rosenfeldt, A. Schenk, T. Scheibel, G. G. Qiao**

Robust β-sheet peptide reinforced polymer fibers

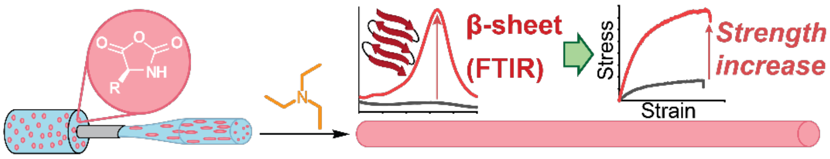

Supporting Information

Robust β-sheet peptide reinforced polymer fibers

Nicholas J. Chan, Sarah Lentz, Paul A. Gurr, Shereen Tan, Mona Schultebeyring, Sabine Rosenfeldt, Anna Schenk, Thomas Scheibel, Greg G. Qiao**

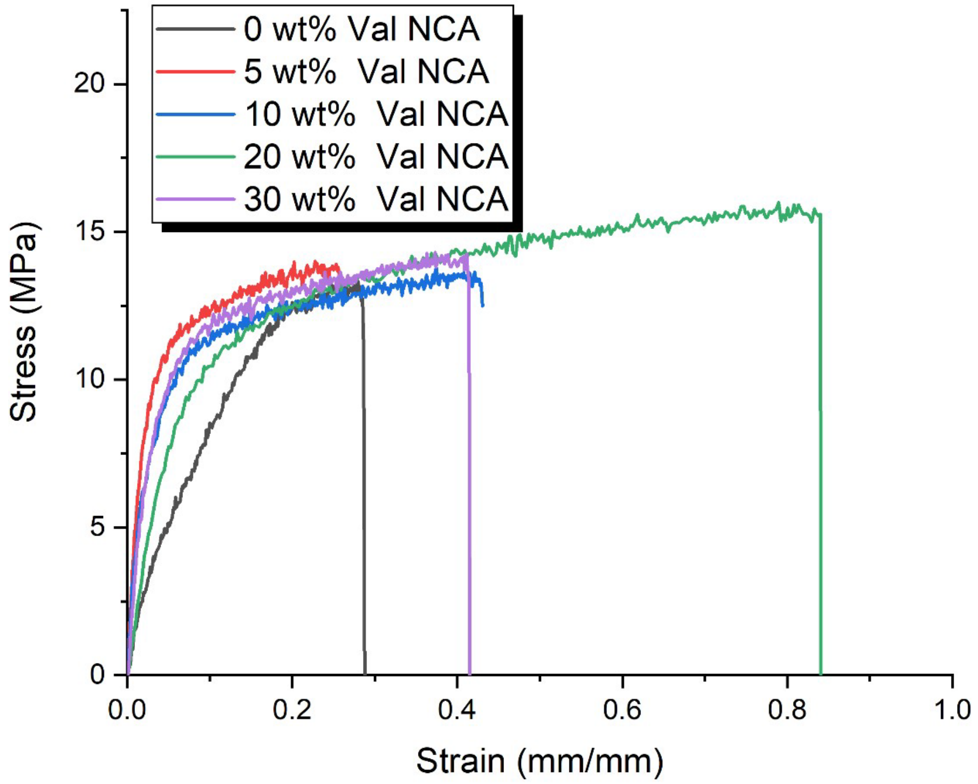

Figure S1: Preliminary mechanical testing of Nylon 6 fibers spun with varying weight fractions of Val NCA

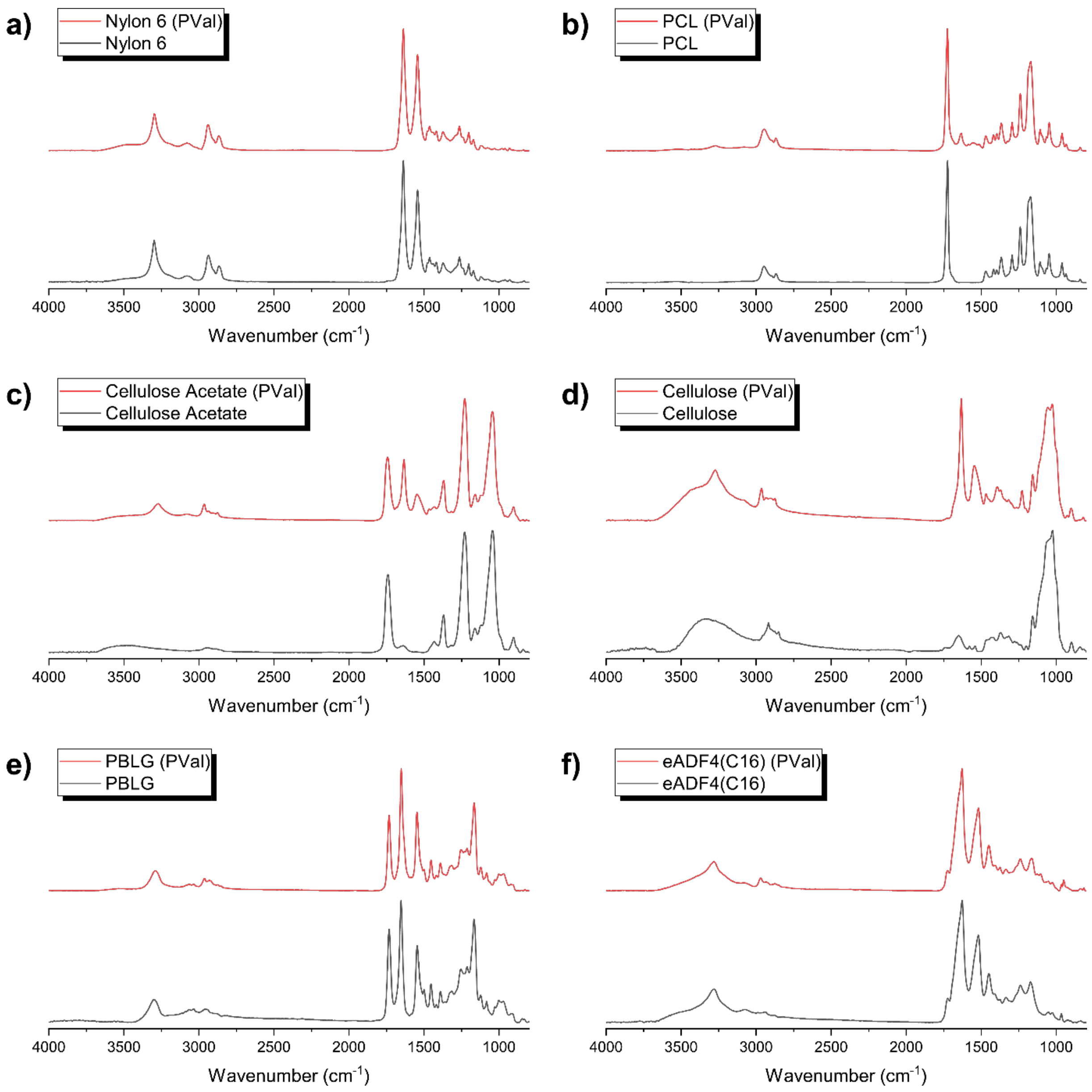

Figure S2: Full Fourier transform infrared (FTIR) spectra for fiber samples in the absence and presence of PVal for a) Nylon 6, b) poly(caprolactone) (PCL), c) cellulose acetate, d) cellulose, e) poly(benzyl-L-glutamate) (PBLG) and f) eADF4(C16).

Table S1: Summary of relative intensity of the β-sheet peak relative to key non β-sheet peaks within each polymer.

**Polymer**	**FTIR band reference peak (cm^{-1})**	**Deconvoluted relative intensity (to 1625-1634 cm^{-1})**	
		Unmodified	With β-sheet
**Nylon 6**	2850 – 2950<br>(CH and CH_2 aliphatic stretch)	1.55 ± 0.03	2.09 ± 0.08

**PCL**	1726 (C=O stretch)	Not present	0.12 ± 0.02
**Cellulose Acetate**	1737-1755 (C=O + O-H stretch)	0.09 ± 0.04	0.44 ± 0.06
**Cellulose**	1740 (O-H stretch)	Not present	0.67 ± 0.14
**PBLG**	1652 (α-helix N-H)	0.02 ± 0.04	0.12 ± 0.02
**eADF(C16)**	1647 (random coil N-H)	1.62 ± 0.07	8.66 ± 2.74

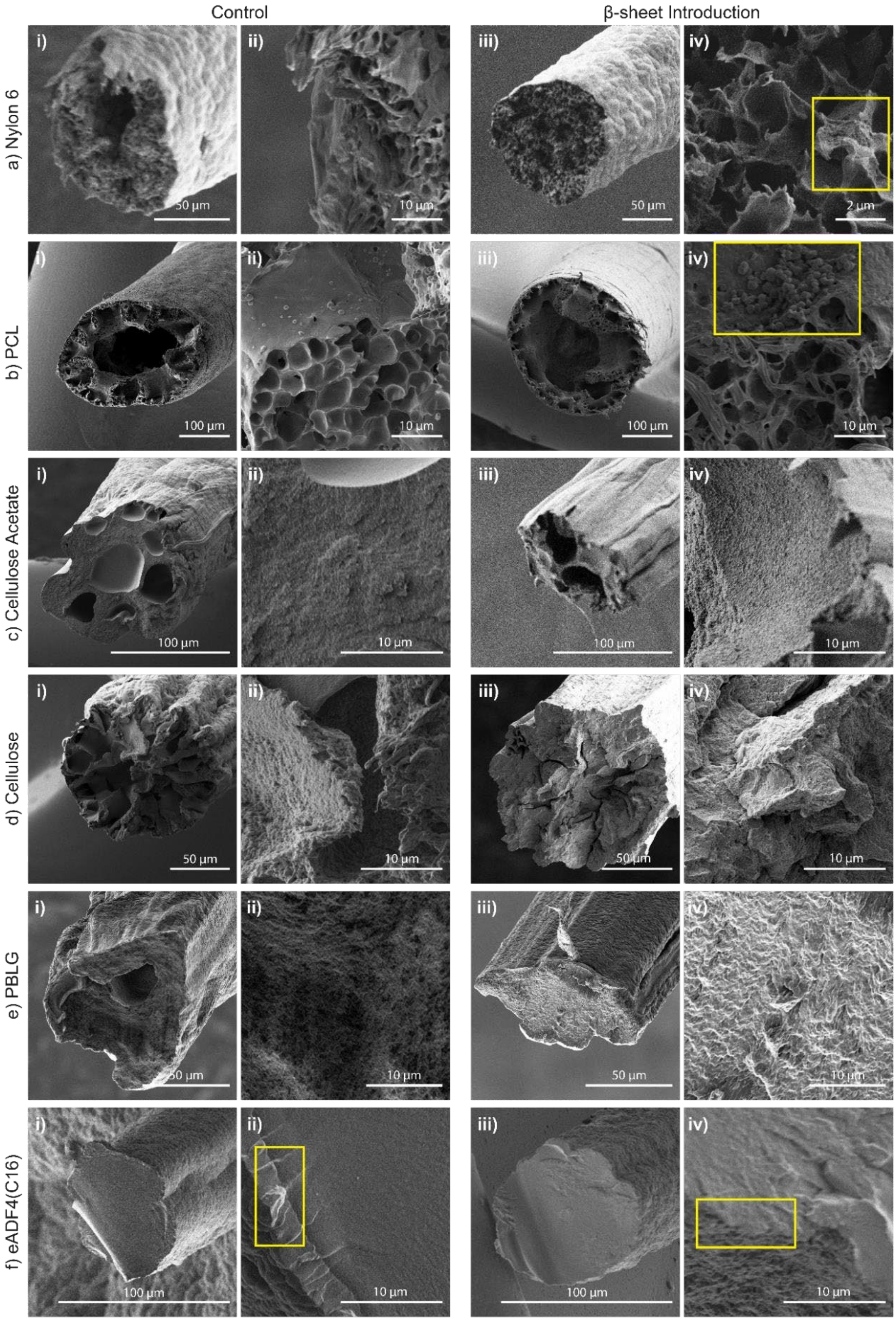

Figure S3: Scanning electron microscopy (SEM) of fiber cross-sections at i, iii) low magnification and ii, iv) high magnification for fiber samples i, ii) in the absence of and iii, iv) in the presence of PVal for a) Nylon 6, b) poly(caprolactone) (PCL), c) cellulose acetate, d) cellulose, e) poly(benzyl-L-glutamate) and f) eADF4(C16). Features mentioned in the text are highlighted in yellow.

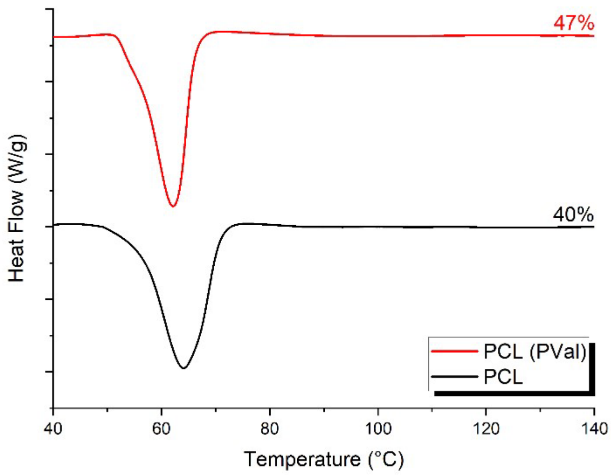

Figure S4: Differential scanning calorimetry (DSC) thermogram of PCL fibers in the absence and presence of PVal with crystallinity quoted

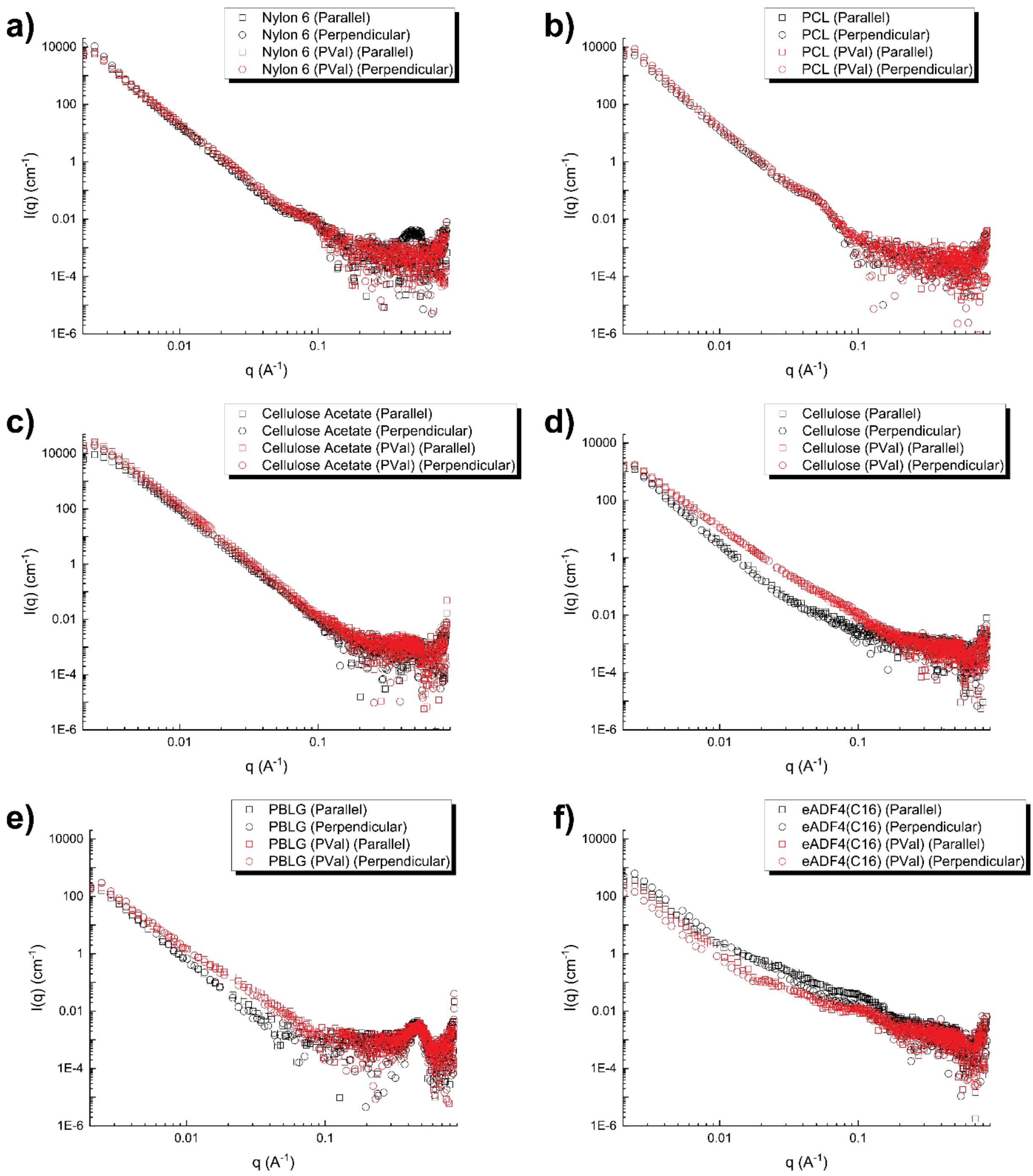

Figure S5: Small angle X-ray scattering (SAXS) spectra for fiber samples with and without PVal for a) Nylon 6, b) poly(caprolactone) (PCL), c) cellulose acetate, d) cellulose, e) poly(benzyl-L-glutamate) (PBLG) and f) eADF4(C16).

Table S2: Summary of mechanical data. All errors represent standard deviation (*n* = 5)

	Strength (MPa)	Extension at Break (mm/mm)	Young's Modulus (MPa)	Toughness (MJ/m^3)

Nylon 6	Without PVal	13.4 ± 0.44	0.318 ± 0.0223	76.7 ± 10.5	4.75 ± 0.55
	With PVal	15.4 ± 0.42	0.909 ± 0.0487	99.4 ± 18.3	16.4 ± 0.71
PCL	Without PVal	0.615 ± 0.06	1.261 ± 0.149	4.98 ± 0.67	0.975 ± 0.18
	With PVal	1.34 ± 0.19	1.40 ± 0.217	12.4 ± 2.26	2.35 ± 0.34
CellAc	Without PVal	36.1 ± 5.21	0.100 ± 0.0120	644.9 ± 128.4	3.94 ± 0.91
	With PVal	154.4 ± 13.4	0.117 ± 0.0162	5014 ± 818.7	19.7 ± 2.54
Cellulose	Without PVal	60.4 ± 4.60	0.185 ± 0.0386	680.3 ± 114.2	11.6 ± 2.63
	With PVal	63.1 ± 3.98	0.162 ± 0.0265	1219.7 ± 263.4	11.8 ±2.07
PBLG	Without PVal	15.5 ± 3.66	0.878 ± 0.0831	164.6 ± 129.6	16.9 ± 3.25
	With PVal	13.1 ± 1.27	1.592 ± 0.287	145.0 ± 12.9	24.9 ± 5.38
eADF(C16)	Without PVal	35.5 ± 2.07	0.0753 ± 0.0051	687.4 ± 140.7	2.47 ± 0.26
	With PVal	18.34 ± 1.44	0.0492 ± 0.0021	677.8 ± 107.3	0.91 ± 0.09
